# Characterization of an Endolysin Targeting *Clostridioides difficile* That Affects Spore Outgrowth

**DOI:** 10.3390/ijms22115690

**Published:** 2021-05-26

**Authors:** Shakhinur Islam Mondal, Arzuba Akter, Lorraine A. Draper, R. Paul Ross, Colin Hill

**Affiliations:** 1 APC Microbiome Ireland, University College Cork, T12 YT20 Cork, Ireland; shakhin200-gen@sust.edu (S.I.M.); l.draper@ucc.ie (L.A.D.); p.ross@ucc.ie (R.P.R.); 2 Genetic Engineering and Biotechnology Department, Shahjalal University of Science and Technology, Sylhet 3114, Bangladesh; 3 Biochemistry and Molecular Biology Department, Shahjalal University of Science and Technology, Sylhet 3114, Bangladesh; arzuba-bmb@sust.edu; 4 School of Microbiology, University College Cork, T12 K8AF Cork, Ireland

**Keywords:** *Clostridioides difficile* infection, endolysin, antimicrobial agent, antimicrobial resistance, enteric pathogen, antibiotics

## Abstract

*Clostridioides difficile* is a spore-forming enteric pathogen causing life-threatening diarrhoea and colitis. Microbial disruption caused by antibiotics has been linked with susceptibility to, and transmission and relapse of, *C. difficile* infection. Therefore, there is an urgent need for novel therapeutics that are effective in preventing *C. difficile* growth, spore germination, and outgrowth. In recent years bacteriophage-derived endolysins and their derivatives show promise as a novel class of antibacterial agents. In this study, we recombinantly expressed and characterized a cell wall hydrolase (CWH) lysin from *C. difficile* phage, phiMMP01. The full-length CWH displayed lytic activity against selected *C. difficile* strains. However, removing the N-terminal cell wall binding domain, creating CWH_351—656_, resulted in increased and/or an expanded lytic spectrum of activity. *C. difficile* specificity was retained versus commensal clostridia and other bacterial species. As expected, the putative cell wall binding domain, CWH_1—350_, was completely inactive. We also observe the effect of CWH_351—656_ on preventing *C. difficile* spore outgrowth. Our results suggest that CWH_351—656_ has therapeutic potential as an antimicrobial agent against *C. difficile* infection.

## 1. Introduction

*Clostridioides difficile* (formerly known as *Clostridium difficile*) is a Gram-positive anaerobic spore-former that is the causative agent of toxin-mediated colitis in humans [1]. In recent decades, *C. difficile* infection (CDI) has become a worldwide health threat that has a high incidence in both hospital and community settings, with increased morbidity and mortality [2]. It has recently been reported that there are nearly 462,100 CDI cases annually in the United States, leading to at least 12,800 fatalities [3,4]. The estimated annual cost of healthcare associated with *C. difficile* infection in the USA is $5 billion and €3 billion in Europe [5,6]. Part of the problem can be attributed to the spores produced by this pathogen that are resistant to some disinfectants, leading to long-term contamination of hospital environments. Disruptions to the gut microbiome, most often because of protracted antibiotic therapy, allows *C. difficile* spore germination, growth, and toxin production in the colon [7]. The pathogenesis of *C. difficile* is typically mediated by two major exotoxins, TcdA and TcdB, that cause intestinal epithelial damage and diarrhoea [8]. Further, spore formation, germination, and outgrowth in *C. difficile* are key elements of CDI transmission and relapse. Recurrency is a common problem in *C. difficile* infection (approximately 60% of patients suffer after three or more infectious episodes) [9,10]. Recurrent CDI has increased over time, with 189% increase in incidence between 2001 and 2012 [11]. A report published in 2019 by CDC concluded that *C. difficile* is among the top three antibiotic-resistant bacteria classified as an urgent threat, and they highlighted the need for development of novel therapeutics and prevention of CDI [3].

Currently, antibiotic therapy remains the treatment of choice for CDI [12]. Oral antibiotics such as metronidazole and vancomycin are commonly prescribed for the first-line treatment of CDI [13]. These two antibiotics significantly impact the commensal gut microbiota and increase the possibility of post-treatment relapse [14]. Another option is fidaxomicin, a narrow-spectrum antibiotic [15]. It is less toxic to obligate anaerobic commensal bacteria and more effective against recurrent *C. difficile* infection, but the high cost associated with this antibiotic limits its clinical use [16]. Other efforts towards the prevention of CDI are faecal microbiota transplantation (FMT), probiotic therapy, and an intravenous infusion of bezlotoxumab (a monoclonal antibody against TcdB). The most effective treatment for recurrent CDI is faecal microbial transplantation. However, donor selection, stool processing, route of administration, timing of transplantation, and screening for potentially transmissible pathogens make this a complicated process [14,17]. Probiotic therapy is promising, but further comprehensive research and studies are essential [18]. Monoclonal antibody treatment is still in clinical trials [19]. Therefore, the development of new drugs is vital. An ideal candidate would be a bactericidal agent with targeted activity against *C. difficile* without causing collateral damage to the indigenous gut microbiota.

Bacteriophages (phages) are viruses of bacteria. Phage therapy involves the targeted application of phages that infect and kill specific pathogenic bacteria [20]. Phage therapy has been used sporadically to eradicate specific bacteria responsible for a wide range of animal and human infections [21,22]. Bacteriophage based therapy offers several advantages over conventional antibiotics, including a narrow spectrum host range [23,24]. While phage therapy has potential, only temperate (lysogenice) *C. difficile* bacteriophages have been isolated to date, limiting the use of phage therapy in CDI [25,26,27]. Bacteriophage-derived lytic enzymes, or endolysins, are potential antimicrobial candidates. Endolysins or lysins rapidly degrade cell wall peptidoglycan (PG) at the end of the lytic cycle to allow for bacteriophage progeny release [28]. Bacteriophage endolysins have been widely investigated as potential antimicrobials via their ability to degrade Gram-positive cell walls when applied externally [29]. Although *C. difficile* lysin research still is in its early stages, it has sparked interest as a therapeutic alternative for CDI [30,31,32,33,34,35]. In the current work, we explored published *C. difficile* genomes and complete bacteriophage sequences that are infecting *C. difficile*. We found a cell wall hydrolase (CWH) lysin encoded by the *C. difficile* phage phiMMP01 that is unique in domain architecture compared to other characterized *C. difficile* lysins. The majority of the reported *C. difficile* lysins are amidases, whereas the CWH comprises two different catalytic domains and one tentative binding domain. We produced truncated constructs and measured their lytic activity in vitro. We conclude that the catalytic domains (CWH_351—656_) were significantly more active than the full-length CWH or any other truncated constructs of CWH. More importantly, CWH_351—656_ inhibits spore outgrowth in vitro, which would be critical in preventing the spread and recurrence of *C. difficile* infection.

## 2. Results and Discussion

### 2.1. In Silico Analysis of C. difficile and Phages Infecting C. difficile in Search of Lytic Enzymes

**Figure 1:**
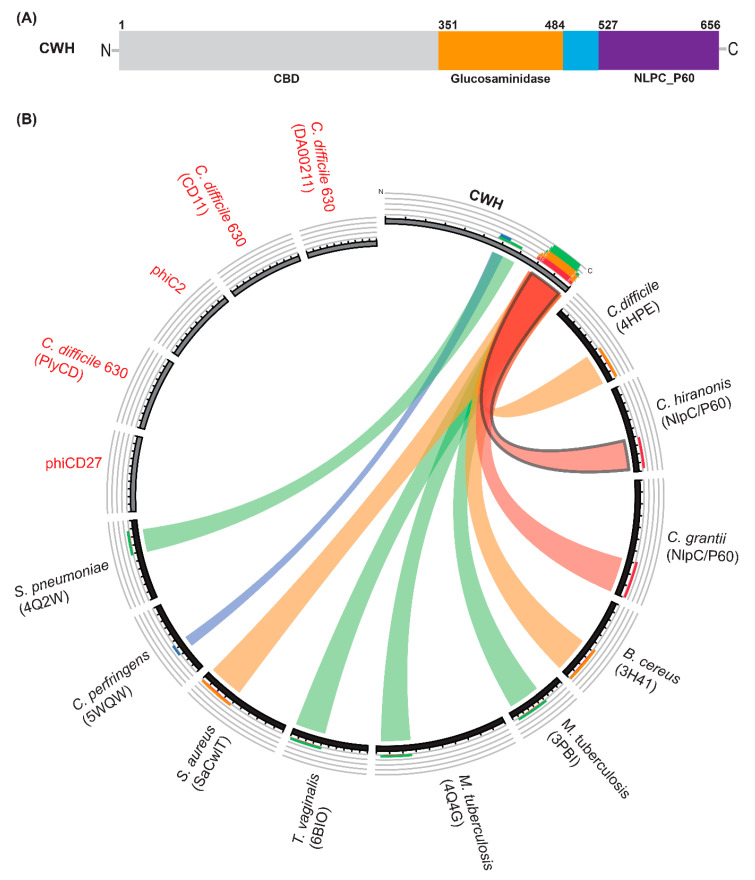
Modular structure and relationship analysis of cell wall hydrolase (CWH). (**A**) Schematic presentation of full-length lysin CWH depicting the putative cell wall binding (CBD) domain (light grey), glucosaminidase domain (orange), and Nlp60 (purple). (**B**) Relationship between CWH and bactericidal enzymes derived from *C. difficile* phages or prophages and other bacterial species visualized by Circoletto software. The ribbons represent the local alignments produced by BLAST (with an E-value cut off 10^−2^), and with colours, blue, green, orange, and red representing bit scores of 25%, 50%, 75%, and 100%, respectively. Previously characterised endolysins from *C. difficile* and phages infecting *C. difficile* are shown here in red text. Protein names shown in parentheses.

We searched published genomes of *C. difficile* and bacteriophages infecting *C. difficile*, and we identified a putative phage lysin (NCBI Reference Sequence YP_009206142.1) from the phage phiMMP01 [36,37] and termed it CWH (cell wall hydrolase). A BLAST search of CWH using non-redundant protein sequences showed similarity with the C40 peptidase family of *C. difficile*. BLAST searches of the protein data bank (PDB) showed some identity (approximately 32 to 41% when the proteins were compared in pairs) to putative cell wall hydrolases encoded by a Tn916-like element in *C. difficile* 630 (4HPE), NlpC/P60 family protein of *Bacillus cereus* (3H41) and *Trichomonas vaginalis* (6BIQ), invasion protein (3PBI), and peptidoglycan endopeptidase (4Q4G) of *Mycobacterium tuberculosis*. Domain identification was carried out using the conserved domain database (CDD) [38], P-FAM [39], and profile-based HHpred server [40] searches indicate that CWH contains two catalytic domains, glucosaminidase and NlpC/P60 (Figure 1A). The N-terminal part of CWH (amino acids 1–350) has no significant similarity with known proteins. However, some similarities were identified with bacteriophage tail proteins using HHpred [40]. We tentatively assigned the N-terminal domain as a putative cell wall binding domain (CBD). The relationship between CWH, phage/prophage endolysins, and bacterial cell-wall modifying enzymes was visualized with Circoletto software [41], available through the Bioinformatics Analysis Team server (http://tools.bat.infspire.org). It is noteworthy that CWH does not resemble any other published characterized endolysin of *C. difficile* (Figure 1B). Bioinformatic analysis revealed similarity between CWH and cell-wall modifying enzymes, NlpC/P60 family protein, and N-acetylglucosaminidases of different bacterial pathogens (Figure 1B). 

### 2.2. Homology Modelling

**Figure 2:**
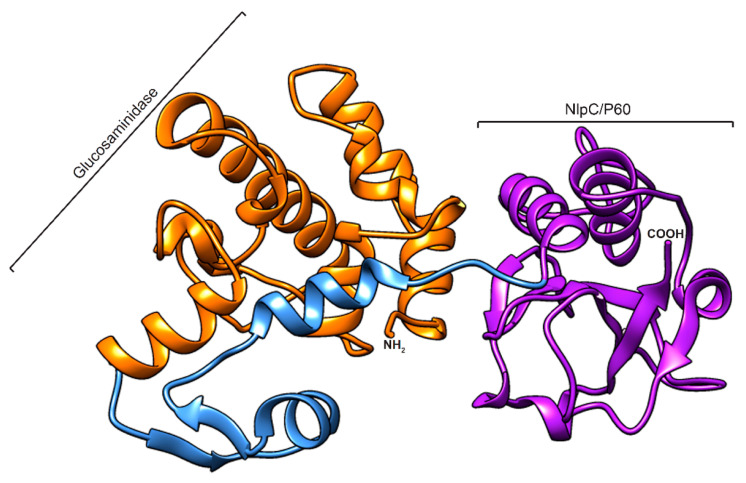
Homology modelled structure of the CWH_351—656_. The 3D structure of the protein is represented as a cartoon, with domain and linkers coloured as follows: orange (glucosaminidase), blue (linker), and purple (Nlp60). The model was generated using the Robetta server and visualized using Chimera software.

As no significant homology to the N-terminal part of CWH (amino acids 1–350) could be found, a three-dimensional homology modelling was only performed on amino acids 351–656 of CWH (see Section 3.3). The predicted 3D structure clearly shows two distinct domains connected by a linker peptide (Figure 2). The predicted model was validated using online quality evaluation tools PROCHECK and ERRAT. The Ramachandran plot (RC plot) of the generated model provided confidence in the model, as the majority of the residues were in the most favourable regions (Figure S1A). The ERRAT quality factor was 95% (Figure S1B), indicating that the model was relevant and reliable.

### 2.3. Expression and Lytic Activity of Endolysins

**Figure 3:**
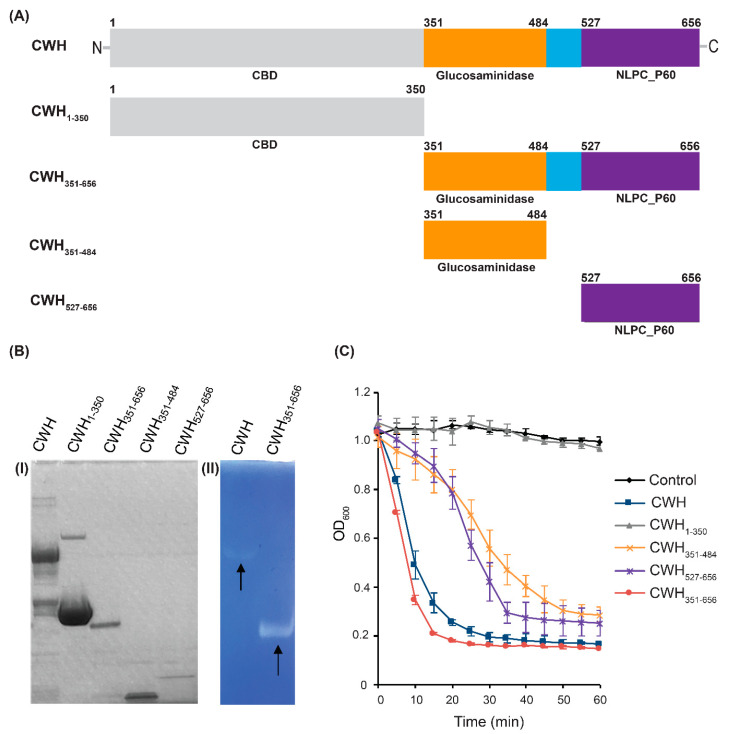
Lytic activity of CWH and its different truncated forms. (**A**) Different truncations of CWH were generated. The numbers above the rectangles correspond to amino acid residue positions. (**B**) (**I**) SDS-PAGE of purified CWH and its different truncated forms. (**II**) Zymogram gel with embedded *C. difficile* cells. Arrows indicating the lytic activity of CWH and CWH_351—656_, seen as a clear zone in the blue background. (**C**) Effect of truncation on lytic activity. Turbidity reduction assays of truncated lysins (200 µg mL^−1^) were performed using *C. difficile* 43255, and error bars represent standard deviations (SD) from duplicate assays.

To assess the roles of the full-length CWH lysin and each of the predicted domains of CWH, different truncated forms of CWH were generated (Figure 3A). The constructs were overexpressed in *E. coli* and purified from the cell lysate proteins as described in the Section 3. The purity and molecular weight were verified on SDS-PAGE (Figure 3B). The relative activity of full-length CWH, putative cell wall binding domain (CWH_1—350_), glucosaminidase domain (CWH_351—484_), Nlp60 domain (CWH_527—656_), and the two catalytic domains together (CWH_351—656_) were analysed using turbidity reduction assays (Figure 3C). All constructs, except for the putative cell wall binding domain, showed lytic activity against *C. difficile*. Full-length CWH and the combined catalytic domains (CWH_351—656_) displayed higher activity than any other construct. Their lytic activity was further confirmed by zymogram assay. Clear lytic zones were observed in the gel, indicating the PG-degrading activity of the lysins (Figure 3B). However, CWH_351—656_ exhibited faster lysis of *C. difficile* cells compared to the full-length CWH lysin (Figure 3C). Generally, the binding domain plays a role in specific binding to cell wall receptors, and the adjacent catalytic domain participates in the cleaving of its substrate. In some lysins, the binding domains are essential for activity [42], whereas others have similar or better lytic activity without the binding domains [43,44,45]. Two previously characterized *C. difficile* lysins CD27L and PlyCD display similar characteristics, with truncated catalytic domains having significantly greater lytic activity [34,35]. Lysins are produced by the phage inside the cell, cleaving the peptidoglycan and releasing the intracellular virus particles. When lysins were applied from the outside, however, it is possible that the outer surface structures of the *C. difficile* cell could hamper peptidoglycan accessibility of the full-length CWH. The secondary structure of cell surface and non-steric cell wall factors may also play a role in preventing access the cell wall [35,46]. Previous studies reported a correlation between the positive charge on lysin’s catalytic domain and its bactericidal activity in the absence of CBD [47,48]. The net positive charge present in CWH_351—656_ could interact more efficiently with the negatively charged bacterial surfaces in the absence of CBD. Such reasoning may explain why CWH_351—656_ exhibits higher and more rapid lytic activity as compared to the full-length CWH. Further experiments were performed using CWH_351—656_.

### 2.4. Characterization of the Catalytic Domains (CWH_351—656_) of Cell Wall Hydrolase (CWH)

**Figure 4:**
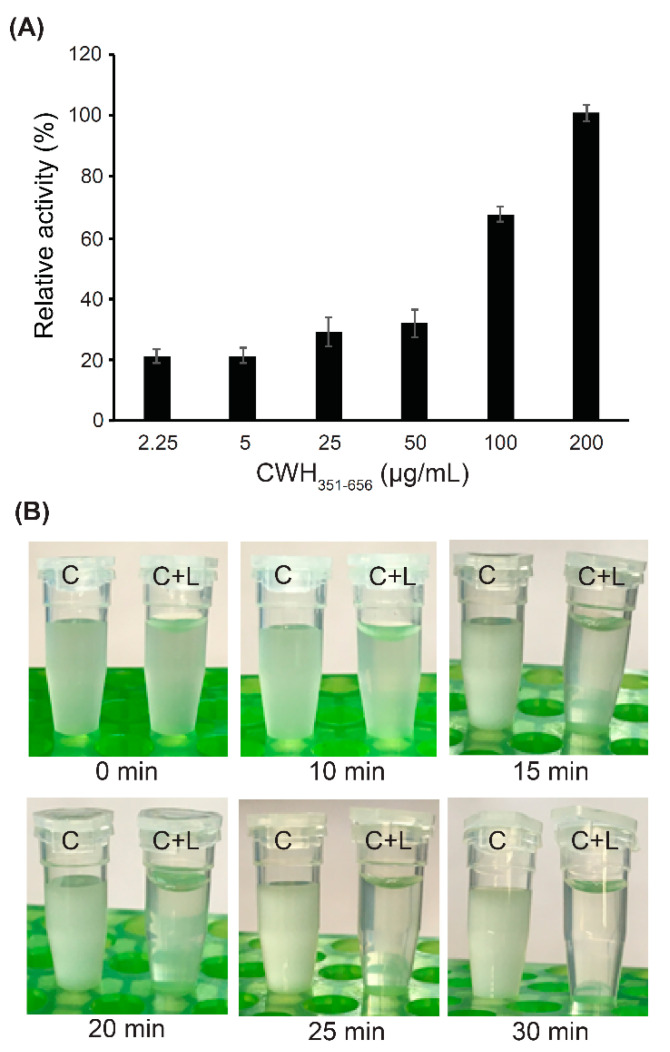
Measurement of CWH_351—656_ lytic activity. (**A**) Relative lytic activity of CWH_351—656_ against *C. difficile*, observed as being dose-dependent. Relative activity of 200 µg mL^−1^ of CWH_351—656_ was determined as 100%. Error bars represent the standard deviations of two independent assays. (**B**) Appearance of *C. difficile* cell suspensions with lysin (C+L) or without lysin (C), photographed over time at room temperature.

Turbidometric analysis provided a quantitative assessment of the lytic activity of CWH_351—656_ by using different concentrations of the lysin (Figure 4A). Addition of 2.25 µg mL^−1^ CWH_351—656_ did not have a strong effect compared to buffer alone; however, gradually higher activities were detected by increasing the lysin protein concentrations. Addition of 200 µg mL^−1^ CWH_351—656_ led to almost complete lysis of *C. difficile* cells. Furthermore, the addition of 200 µg mL^−1^ CWH_351—656_ to *C. difficile* cell suspensions began to clear the culture within 15 min with complete clearing achieved within 30 min (Figure 4B). In contrast, the *C. difficile* cell suspensions without lysin treatment exhibited no changes in turbidity within the 30 min time period (Figure 4B). This rapid and pronounced clearing of *C. difficile* cell suspensions indicate the promising antimicrobial effect of CWH_351—656_. 

**Figure 5:**
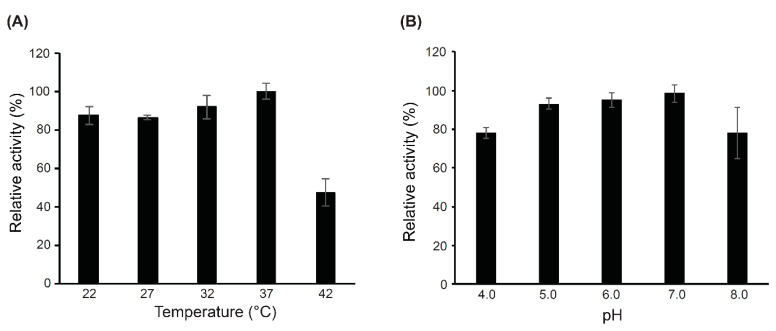
Identifying the optimal temperature and pH for CWH_351—656_ lytic activity. (**A**) The effects of temperature on the lytic activity of CWH_351—656_. The stability was tested by heating the protein at different temperatures (22–42 °C) for 60 min using *C. difficile* cells. (**B**) Influence of pH on the bactericidal activity of CWH_351—656_ incubated with PBS buffer with different pH (4.0–8.0) values. Relative lytic activity was obtained by comparing the lytic activity of each test with the maximal lytic activity among the dataset. Each column represents the mean of duplicate experiments, and error bars indicate the standard deviation.

The optimal temperature and pH conditions for the bactericidal activity of CWH_351—656_ were tested by assessing turbidity reduction in *C. difficile* suspensions at OD_600_ over 60 min. As shown in Figure 5A, CWH_351—656_ was active across a broad range of temperatures between 22 and 37 °C. Optimal activity was observed at 37 °C, but the activity decreased dramatically at 42 °C. CWH_351—656_ was active from pH 4.0 to 8.0, with pH 7.0 observed to be optimal (Figure 5B). The results indicate that CWH_351—656_ has good pH and temperature stability and activity, a favourable feature for an antimicrobial agent that would be deployed in the gut. 

### 2.5. Host Specificity of Full-Length CWH and CWH_351—656_

Although some lysins exhibit broad host range activity [49], most lysins kill specific bacterial taxa [32]. The antimicrobial activity of the full-length CWH and CWH_351—656_ were evaluated against Gram-positive bacteria, including different *C. difficile* strains, *Clostridium* species, and non-*Clostridium* species (Table 1). All species were cultured until mid-exponential phase, washed, and resuspended in PBS at pH 7.0. After mixing with lysins, the OD_600_ values of each culture were recorded over 60 min. CWH_351—656_ retained the same specificity as the full-length CWH when tested against different *C. difficile* strains, including two clinical strains (APC1401 and APC1412). However, the full-length CWH showed only moderate lytic activity against *C. difficile* 630 and *C. difficile* APC 1401, as compared to CWH_351—656_ that showed strong lytic activity against these strains. This indicates that CWH_351—656_ has broader lytic activity against a wider spectrum of *C. difficile* strains. Both CWH and CWH_351—656_ lacked lytic activity against two other clostridia species tested, *C. perfringens* and *C. symbiosum*. However, CWH and CWH_351—656_ exhibited low and moderate lytic activity against *C. scindens*. To determine if full-length CWH and CWH_351—656_ are effective against other non-*Clostridium* species, strains of *Bacillus*, *Enterococcus*, *Lactobacillus*, *Lactococcus*, *Listeria*, *and Staphylococcus* were also tested. *B. cereus* was sensitive to lysis by CWH, with CWH_351—656_ showing greater activity. Furthermore, CWH_351—656_ showed low lytic activity against *E. faecalis* and *L. innocua*. The catalytic domains present in CWH include glucosaminidase and Nlp60. The glucosaminadases cleave the glycosidic bond of the sugar backbone, whereas Nlp60 has a diverse range of catalytic activity including cleavage of the *N*-acetylmuramate-l-alanine linkages and the 4–3 linkages between d-Glu and *m*-DAP residues [32]. *C. difficile* and some other species that were sensitive to lysis with CWH and CWH_351—656_ all possess peptidoglycan type A1γ, where a meso-diaminopimelic acid (meso-A2pm) residue at position 3 of the peptide is directly cross-linked to a D-alanine at position 4 of the neighbouring peptide [50]. However, other species described as having the same type of peptidoglycan structure appeared to be resistant to lysis. This variation of specificity may be due to other factors, for example, the sugar backbone has been shown to differ between species [51], also the structural requirement for sensitivity may be different [34]. Moreover, accessing the peptidoglycan from the outside of the cell may be affected by the presence of other cell wall structures. Although CWH_351—656_ showed a higher level of activity against some other commensal gut microbiota tested, removal of the cell wall binding domain did not greatly increase the host range. The truncation of other described *C. difficile* endolysins has also resulted in a wider activity spectra [34,35]. The strong lytic activity of CWH_351—656_ against different ribotypes and toxin types of laboratory reference and clinical *C. difficile* strains is crucial in suggesting it has promise as a suitable antimicrobial candidate.

### 2.6. Optical Microscopy of Lytic Activity

**Figure 6:**
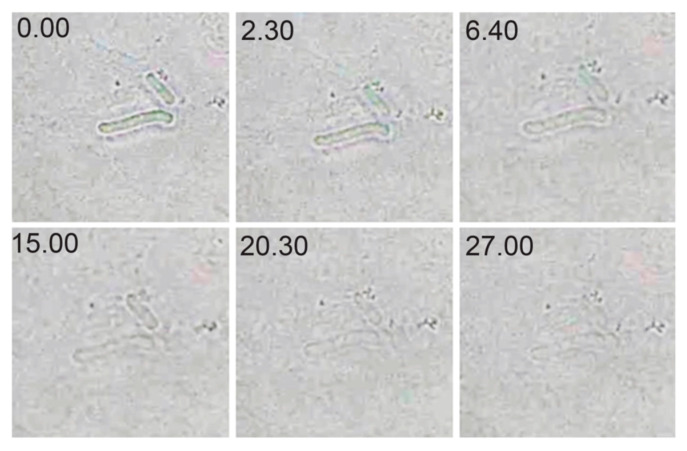
Optical microscopy of the action of CWH_351—656_ on *C. difficile*. Cells are mixed with CWH_351—656_ and were sequentially photographed at subsequent time intervals until complete lysis was observed (27 min).

The lytic activity of CWH_351—656_ was visualized using optical microscopy with exponentially growing *C. difficile* cells treated with the lysin at its optimal pH (pH 7.0) and room temperature (Figure 6). Lysis was recorded within 7 min, and complete lysis was observed at 27 min. These observations are consistent with the optical drop and turbidity observation assays. 

### 2.7. Effect of CWH_351—656_ on C. difficile Spore Outgrowth

**Figure 7:**
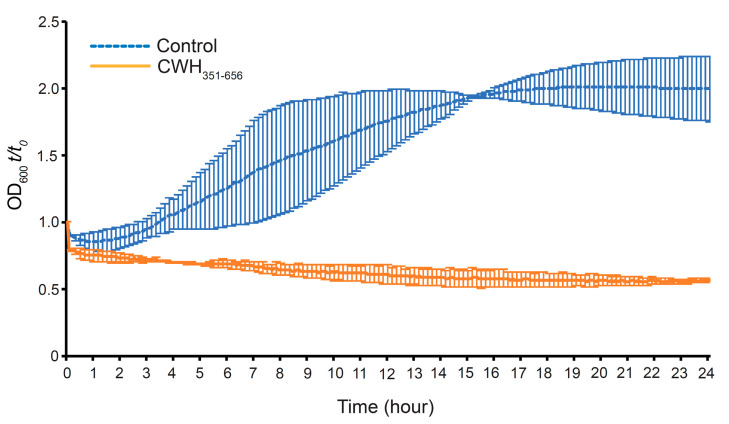
Effect of lysin CWH_351—656_ on germination and outgrowth of *C. difficile* spores. Purified spores, with heat activation, containing 10^7^ spores/mL were added to pre-reduced BHI supplemented with 0.1% sodium taurocholate and mixed with 200 µg mL^−1^ CWH_351—656_ inside an anaerobic chamber. Spore germination and outgrowth were monitored by measuring the OD_600_ of the culture and expressing it as a percentage of the initial OD_600_ (*t*/*t*_0_). Germination was measured as the initial loss of OD_600_, and spore outgrowth was measured by recording the increase in OD_600_ following spore germination. Values are averages ± standard deviations for duplicate determinations.

The effect of CWH_351—656_ on *C. difficile* spore outgrowth was observed by taking OD_600_ values over a 24h time period. Spores were isolated using Clospore medium (see Section 3) from *C. difficile* stains APC43 and APC1401. On the basis of spore quality, we selected APC1401 spores for further experimentation. Spore outgrowth was observed in untreated control samples, as indicated by an increase in OD_600_ starting at 2h (Figure 7). However, the CWH_351—656_ treated (200 µg mL^−1^) sample completely inhibited spore outgrowth, as no increase in absorbance was observed. The CWH_351—656_ treated sample displayed an initial decline in the OD_600_ compared to the control sample, indicating possible inhibition of spore germination. These results suggest that CWH_351—656_ inhibits *C. difficile* vegetative cells growth from newly germinated spores. This is notable, as inhibition of spore gemination and outgrowth could have clinical relevance, minimising the transmission and relapse of CDI [52,53].

## 3. Materials and Methods

### 3.1. Bacterial Strains and Growth Conditions

*C. difficile* strains APC43 (alternative name ATCC 43255 and ribotype 087, a high-level toxin-producing strain isolated from an abdominal wound), *C. difficile* 630 (ribotype 012), *C. difficile* APC1401 (Ribotype R126, isolated from cystic fibrosis patient stool), and *C. difficile* APC1412 (Ribotype R046, isolated from cystic fibrosis patient stool) were obtained from APC Microbiome Ireland’s culture collection. *C. difficile* 28196, *Clostridium scindens* 5676, and *Clostridium symbiosum* 934 were purchased from DSMZ. The other strains used in this study were *Bacillus cereus* DPC 6087, *Clostridium perfringens*, *Enterococcus faecalis* DPC 5152, *Enterococcus faecium* DPC 5137, *Lactobacillus paracasei* 338, *Lactococcus lactis* MG1363, *Listeria innocua*, *Listeria monocytogenes* 1028, and *Staphylococcus aureus* DPC 5247 were obtained from the APC Microbiome Ireland culture collection. All strains were stored at −80 °C and cultivated at 37 °C. *Clostridioides* and *Clostridium* strains were grown anaerobically in BHIS medium (Oxoid brain heart infusion (BHI) supplemented with yeast extract (0.5%, wt/vol) and L-cysteine (10%, wt/vol)). *Escherichia coli* was grown in Luria-Bertani (LB) broth shaken at 37 °C. *Staphylococcus*, *Listeria*, *Enterococcus*, *Pseudomonas*, *Bacillus*, and *Lactococcus* strains were cultivated in BHI broth. *Lactobacillus* strains were cultivated in de Mas, Rogosa, and Sharpe (MRS) broth (Oxoid). 

### 3.2. Cloning, Expression, and Purification of CWH and Its Subdomains

The gene sequence of CWH within the genome of *C. difficile* phage phiMMP01 was obtained from the NCBI database (NCBI Reference Sequence YP_009206142.1) and optimized by Gen Script codon optimization software (http://www.genscript.com/) for *E. coli* expression. The sequence was synthesized and inserted into the pET28b(+) vector by GenScript. The putative cell wall binding domain (CWH_1—350_), glucosaminidase domain (CWH_351—484_), Nlp60 domain (CWH_527—656_), and glucosaminidase and Nlp60 domains together (CWH_351—656_) were produced by PCR from GenScript synthesized CWH-pET28b(+) plasmid. The primers used for PCR amplification are listed in Table S1. After amplification, the products were subcloned into pET28b(+). All pET28b(+) constructs were transformed into *E. coli* TOP10 cells for sequence confirmation. The pET28b(+) plasmids were then introduced into *E. coli* BL21 (DE3) cells, which were grown at 37 °C in LB containing kanamycin (50 µg/mL). The culture was then induced for 2–3 h with 0.1 mM IPTG once the OD_600_ reached 0.6–0.8. Cells were then pelleted, resuspended in binding buffer (50 mM sodium phosphate pH 7.4, 300 mM sodium chloride, 10 mM imidazole), and lysed by FastPrep (MP Biomedicals, Solon, OH, USA) or sonication. Cells were pelleted by centrifugation and purification with His-Spin Protein Miniprep™ Columns (Zymo Research, Irvine, CA, USA). The purified lysins were analysed by SDS-PAGE.

### 3.3. Structural Modelling of CWH_351—656_

To identify the optimal template for homology modelling, the amino acid sequence of CWH_351—656_ was submitted to the HHPred server [40]. The closest phylogenetic relative whose model is well characterized in the RCSB Protein Data Bank (PDB) [54] was selected from the resulting HHPred hit list based on the percent of identity. The putative cell wall hydrolase (PDB ID: 4HPE) from *C. difficile* 630 was selected with 25% identity (E-value = 5.9 × 10^−10^). Homology modelling was carried out using the Robetta Server (http://robetta.bakerlab.org/) to design the three-dimensional (3D) structure. The predicted model was subjected to energy minimization and refinement using ModRefiner [55]. The stereochemical quality of the predicted model was assessed using PROCHECK [56] and ERRAT [57]. Finally, the 3D structure was visualized using Chimera software [58].

### 3.4. Lytic Activity Assays

Turbidity reduction assays were conducted as previously described [35] to analyse the lytic activities of *C. difficile* phage lysin against *C. difficile* strains and other bacterial species. Briefly, under anaerobic conditions, *C. difficile* strains were grown to mid-log phase, and cells were collected by centrifugation (3000× *g* for 5 min). The bacterial pellets were washed twice and resuspended in phosphate-buffered saline (PBS). Before performing analysis, the optical density at 600 nm (OD_600_) was adjusted to approximately 0.9–1.1. The final concentration of 200 µg mL^−1^ of lysin was added for the lytic activity assay. The reduction in OD_600_ was measured every 5 min for 60 min using a microplate reader (Thermo Fisher, Waltham, MA, USA). Lytic activities were also detected by zymography as described previously [28,59] using SDS-polyacrylamide gels containing heat-killed *C. difficile* cells.

To determine the effect of temperature on lysin activity, the same experimental conditions and turbidity reduction assays described above were performed using *C. difficile* strain APC43 at the following temperatures: 22, 27, 32, 37, and 42 °C. To determine the optimal pH for lysin, turbidity reduction assays were performed using strain APC43 in buffers of pH 4.0, 5.0, 6.0, 7.0, and 8.0. 

### 3.5. Optical Microscopy of Lytic Activity

Exponentially growing *C. difficile* APC43 cells were washed twice in PBS by centrifugation and resuspension in the same buffer. Subsequently, the cell pellet was mixed with CWH_351—656_ to a final concentration of 200 µg mL^−1^. The mixture was immediately transferred from an agarose pad (2% dissolved in 20 mM PBS (pH 7.0)) to a cover glass for imaging. Images were captured immediately using the Leica AirLab App for Leica Microsystem (Model ICC50 W) and using 1000x magnification.

### 3.6. C. difficile Spore Preparation

*Clostridioides difficile* spores were prepared as previously described, with slight modifications [60]. Briefly, single colonies of *C. difficile* APC1401 were separately inoculated into BHIS medium and cultured overnight at 37 °C under anaerobic conditions. The next day, 20 mL culture was transferred into 500 mL of Clospore medium [61] and grown for 5–7 days at 37 °C anaerobically. Spores were collected by centrifugation at 1500× *g* for 20 min at 4 °C and washed with cold, sterile, distilled water 3–5 times to ensure spore purity. Spore stocks were stored at 4 °C in sterile water until use. Viable spores were enumerated by plating for colony-forming units (c.f.u.) mL^−1^ on BHIS agar plate supplemented with 0.1% sodium taurocholate (Sigma).

### 3.7. Effect of CWH_351—656_ on C. difficile Spore Germination and Outgrowth

The effect of CWH_351—656_ on *C. difficile* spore germination was determined by adding 25 µL of a suspension containing 10^7^ spores mL^−1^ to the wells of a 96-well plate containing 250 µL of pre-reduced BHIS supplemented with 0.1% sodium taurocholate (Sigma). To this 200 µg mL^−1^ of lysin was added inside the anaerobic workstation. BHIS without spore suspensions or spore suspensions alone were included as controls. For monitoring the anaerobic environment, 0.1 mg mL^−1^ resazurin was included in one well. The plate was sealed with a sealant inside the anaerobic chamber. The microplate reader (Thermo Fisher, Waltham, MA, USA) was used to monitor the OD_600_ for 24 h in 5 min time intervals and was expressed as a percentage of the initial OD_600_ (*t*/*t*_0_). The initial loss of OD_600_ indicates the spore germination and spore outgrowth was measured by the increase in OD_600_ following spore germination, as mentioned previously [62,63].

## 4. Conclusions

*C. difficile* infection has emerged as a worldwide major health problem. Antibiotics disrupt the normal gut microbiota by altering its composition and the gut’s metabolic functions, decreasing its colonization resistance and increasing the risk of CDI. Development of alternative treatment strategies for CDI is currently receiving increasing attention. Bacteriophage-derived lysins are of increasing interest as antibacterial agents, especially against multi-drug resistant pathogens. We have exploited the modular structure of a cell wall hydrolase lysin derived from a *C. difficile* phage. In vitro tests suggest this lysin has potential in the control of CDI. We report that a truncated lysin consisting of the two catalytic domains, CWH_351—656_, displayed faster lytic action against *C. difficile*. Most importantly, CWH_351—656_ can inhibit *C. difficile* spore outgrowth. The results of the current study indicate that CWH_351—656_ is a promising therapeutic option against *C. difficile*, effective in targeting growth and preventing spore outgrowth. In vivo studies using a valid animal model are warranted to confirm the findings presented in this study.

## Figures and Tables

**Table 1:** Antimicrobial activity of Cell Wall Hydrolase (CWH) and CWH_351—656_ on Gram-positive species.

*Clostridioides* Strains	Lysin Activity ^#^	
	CWH	CWH_351—656_
*Clostridioides difficile* 43255	+++	+++
*Clostridioides difficile* APC 1401	++	+++
*Clostridioides difficile* 630	++	+++
*Clostridioides difficile* APC 1412	+++	+++
*Clostridioides difficile* 28196	+++	+++
**Non-*Clostridioides* Strains**		
*Bacillus cereus*	+	++
*Clostridium perfringens*	–	–
*Clostridium scindens*	+	++
*Clostridium symbiosum*	–	–
*Enterococcus faecalis*	–	+
*Enterococcus faecium*	–	–
*Lactobacillus paracasei*	–	–
*Lactococcus lactis*	–	–
*Listeria innocua*	–	+
*Listeria monocytogenes*	–	–
*Staphylococcus aureus*	–	–

^#^ Degree of OD reduction; >0.6 +++, 0.4 to 0.6 ++, 0.2 to 0.4 +, <0.2 –.

## Data Availability

The data presented in this study are available on request from the corresponding author.

## Supplementary Materials

The following are available online at https://www.mdpi.com/article/10.3390/ijms22115690/s1.
