# Supplementary material for: Characterization of an Endolysin Targeting Clostridioides difficile That Affects Spore Outgrowth
Source: Int J Mol Sci. 2021 May 26;22(11):5690. doi: 10.3390/ijms22115690 (PMC8199566; doi:10.3390/ijms22115690)
Supplement: Supplementary file 1 [file ijms-22-05690-s001.zip › ijms-1197637-supplementary/Supplementary Figure 1.pdf]

(A)

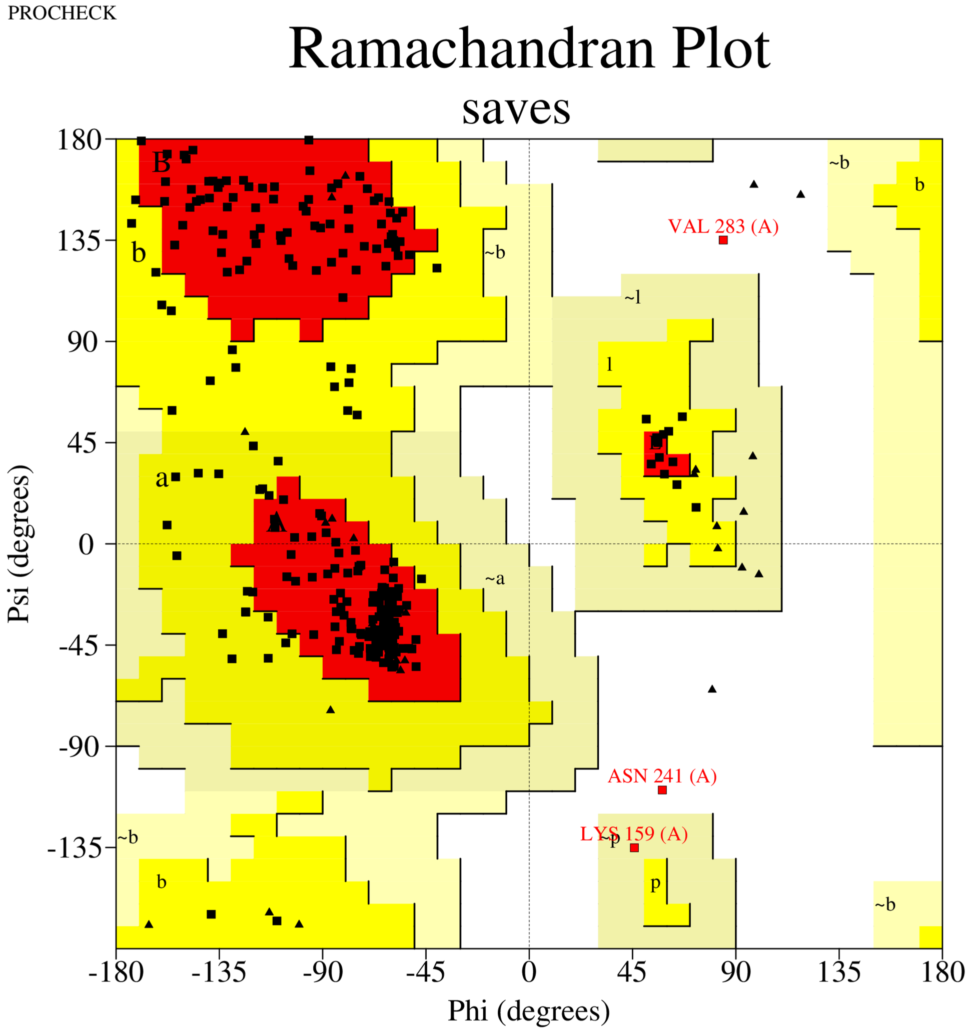
PROCHECK
Ramachandran Plot
saves
180
135
90
45
0
-45
-90
-135
Psi (degrees)
-180
-135
-90
-45
0
45
90
135
180
Phi (degrees)
B
b
a
~b
~l
l
~a
~p
p
VAL 283 (A)
ASN 241 (A)
LYS 159 (A)

(B)

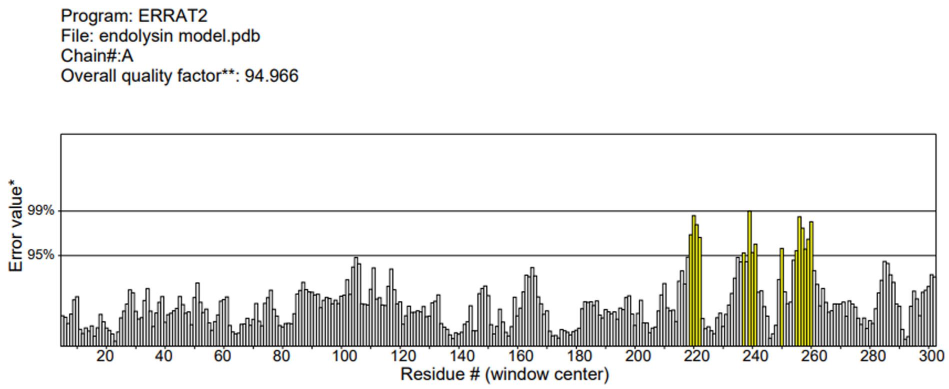
Program: ERRAT2
File: endolysin model.pdb
Chain#:A
Overall quality factor**: 94.966
Error value*
99%
95%
20
40
60
80
100
120
140
160
180
200
220
240
260
280
300
Residue # (window center)
