# Supplementary material for: Characterization of an Endolysin Targeting Clostridioides difficile That Affects Spore Outgrowth
Source: Int J Mol Sci. 2021 May 26;22(11):5690. doi: 10.3390/ijms22115690 (PMC8199566; doi:10.3390/ijms22115690)
Supplement: Supplementary file 1 [file ijms-22-05690-s001.zip › ijms-1197637-supplementary/Supplementary Table 1.pdf]

Supplementary Table 1: Primers used for PCR amplification of different construct of CWH

Primers	[a]Sequences (5′–3′)
CWH_Glu_F	AACGGATCC**ATG**AACGGTCCGGCGCAA
CWH_Glu_R	CGCAAGCTT**TTA**GATCTGCTTGTAGAA
CWH_Nlpc_F	AACGGATCC**ATG**AACAACAAAGTGAGC
CWH_Nlpc_R	CGCAAGCTT**TTA**AAAGAAACGACGCGC
CWH_CBD_F	CGGGATCCG**ATG**CTGAGCGACGATCTG
CWH_CBD_R	CGCAAGCTT**TTA**CTGCTTCTCGTTTTT

[a]Restriction sites are indicated by underlining. Start and stop codons are indicated by bold letters
